# Microtubule Stabilization Promotes Microcirculation Reconstruction After Spinal Cord Injury

**DOI:** 10.1007/s12031-020-01679-5

**Published:** 2020-09-08

**Authors:** Yang-Yang Duan, Yong Chai, Nai-Li Zhang, Dong-Mei Zhao, Cheng Yang

**Affiliations:** Department of Anatomy, Binzhou Medical University, Yantai, 264003 Shandong China

**Keywords:** Endothelial cells, Microcirculation, Microtubule, Pericytes, Spinal cord injury

## Abstract

Spinal cord microcirculation plays an important role in maintaining the function of spinal cord neurons and other cells. Previous studies have largely focused on the ability of microtubule stabilization to inhibit the fibroblast migration and promote axon regeneration after spinal cord injury (SCI). However, the effect of microtubule stabilization treatment on microcirculation reconstruction after SCI remains unclear. By using immunofluorescence, we found that microtubule stabilization treatment improved microcirculation reconstruction via increasing the number of microvessels, pericytes, and the perfused microvessels after SCI. To clarify the underlying mechanisms, rat brain microvascular endothelial cells and pericytes were subjected to glucose oxygen deprivation. By using flow cytometry and western blotting, we found that microtubule stabilization treatment inhibited apoptosis and migration of endothelial cells and pericytes but promoted proliferation and survival of endothelial cells and pericytes through upregulated expression of vascular endothelial growth factor A (VEGFA), VEGF receptor 2, platelet-derived growth factor-B (PDGFB), PDGF receptor β, and angiopoietin-1 after SCI. Taken together, this study provides evidence for the mechanisms underlying the promotion of microcirculation reconstruction after SCI by microtubule stabilization treatment. Importantly, this study suggests the potential of microtubule stabilization as a therapeutic target to reduce microcirculation dysfunction after SCI in the clinic.

**Electronic supplementary material:**

The online version of this article (10.1007/s12031-020-01679-5) contains supplementary material, which is available to authorized users.

## Introduction

Spinal cord injury (SCI) often leads to the lifelong sensorimotor and autonomic neurological dysfunctions in the patient. The poor prognosis creates a heavy burden on both the family and society (Qiu 2009). Owing to the lack of the efficient treatment, the functional recovery after SCI remains challenging (Fitch and Silver 2008). The repairing mechanisms and functional recovery have been extensively studied. Different approaches have been tried in the past decades, for example, stem cell transplantation, tissue engineering, and gene therapy (Varma et al. 2013). However, there is still no effective method to improve functional recovery after SCI.

After SCI, the local vasculature in the injured site is disrupted. Subsequent pathological changes including endothelial cell and pericyte loss, vessel density decrease, and an organization disorder that contribute to the spread and aggravation of secondary inflammation damages to the central nervous system have been reported (Mautes et al. 2000). The breakdown of microvessels promotes the entry of inflammatory cells and cytokines into the nervous system through the injury site to exacerbate neurological dysfunction. Moreover, the dysfunctional microvessels lead to apoptosis in other cells in the central nervous system, such as astrocytes, microglia, and neurons (Ling and Liu 2007; Liu et al. 1997). Recent researches have shown that the regenerating axons tend to grow in close interaction with blood vessels, which provides them with a nutritionally favorable environment for rapid growth, and that the endothelial cells could engulf myelin debris to boost the recovery after SCI (Dray et al. 2009; Zhou et al. 2019). Poor microvascular perfusion leads to dorsal column sensory axon degeneration after SCI in rats (Muradov et al. 2013). Therefore, the microvessel regeneration is beneficial for recovery after SCI. It has been reported that cell shape stabilization and migration adapting to the various driving forces orchestrated by the microtubules are key steps of angiogenesis (Bayless and Johnson 2011). In angiogenesis initiation, the vessel growth direction is established by the microtubule. In blood vessel formation, vesicle/vacuole trafficking along the microtubule cytoskeleton in endothelial cells appears to form tubes and provide nutrition, which depends on microtubule stabilization (Norden et al. 2016). Intact and stabilized microtubules are also crucial for maintaining the newly formed vessels (Bayless and Davis 2004; Norden et al. 2016). Furthermore, a recent study indicated that the microtubule stabilization in endothelial cells led to lumenogenesis and sufficient tissue reperfusion after ischemic injury (Li et al. 2017a).

Recently, Ruschel has demonstrated that systematic administration of epothilone B (Epo B) promotes functional recovery through microtubule stabilization after SCI (Ruschel et al. 2015). Importantly, Epo B can permeate the blood-brain barrier (Ballatore et al. 2012). Microtubule stabilizing drugs promote motor function recovery by interfering with fibroblast migration to reduce fibrotic scar and by acting on the microtubule associated protein Tau to improve intrinsic neuron growth (Ruschel and Bradke 2018; Ruschel et al. 2015; Sandner et al. 2018).

However, the role and mechanism of microtubules on the restoration of vasculature after SCI remains unclear. In this study, we investigated potential effects of stabilized microtubules in microcirculation reconstruction after SCI and their underlying mechanisms. Our results revealed that microtubule stabilization promoted angiogenesis to improve prognosis after SCI. Importantly, the microtubule stabilizing treatment may present as a novel strategy for the treatment of SCI in the clinic.

## Materials and Methods

### Cell culture

Rat brain microvascular endothelial cells and pericytes were obtained from iCell Bioscience Inc (Shanghai, China, RAT-iCELL-n001, RAT-iCELL-n003). Endothelial cells were cultured in Endothelial Cell Medium (10% fetal bovine serum and 1% endothelial cell growth supplement, PriMed-iCELL-002) and kept in a humidified incubator (37 °C, 5% CO_2_, and 95% O_2_). Passages between 1 and 4 were used in all experiments. Pericytes were cultured in Pericyte Medium (2% fetal bovine serum and 1% pericyte growth supplement, PriMed-iCELL-015) and kept in a humidified incubator (37 °C, 5% CO_2_, and 95% O_2_). Passages between 2 and 4 were used in all experiments.

### Oxygen and glucose deprivation (OGD) model in vitro

To mimic the ischemic and hypoxic conditions after SCI, endothelial cells and pericytes were cultured in a hypoxic condition for 15 min, 30 min, and 60 min. The hypoxic condition was produced by replacing normal medium with the serum-free medium without glucose (Cat. 11966-025, Gibco) and placing the plates and petri dishes in a hypoxic incubator (Modular Incubator Chamber, Brincubator). The hypoxic incubator was filled with 95% N_2_ and 5% CO_2_ for 8 min before use.

### Extraction of tubulin fractions

The free and polymerized tubulin fractions were isolated using a method previously described by Li et al (Li et al. 2015). Cells (2 × 10^6^) grown in 35-mm petri dish plates were washed twice with a microtubule stabilization buffer (MTSB, 0.1 M piperazine-N, N′-bis (PIPES), 2 mM ethylene glycol-bis (*β*-aminoethylether) *N,N,N*′*,N*′-tetraacetic acid (EGTA), 2 mM ethylenediaminetetraacetic acid (EDTA), 0.5 mM MgCl_2_, and 2 M glycerol). The cells were incubated with MTSB + 0.1% Triton X-100 + protease inhibitor cocktail (1:200) for 30 min, and the supernatant was collected as the free tubulin fraction. The Triton X-100 insoluble fraction, corresponding to the polymerized tubulin, was then solubilized in a RIPA lysis buffer (Dalian Meilun Biology Technology Co., Ltd., China) with a protease inhibitor cocktail. Cells were lysed for 30 min and centrifuged at 15,000*g* for 15 min at 4 °C. The polymerized and free tubulin fractions were quantified using western blotting analysis.

### Western blotting

Free and polymerized tubulin fractions prepared as described above were probed with anti-α-tubulin (Abcam, ab52866, 1:5000), GAPDH (Biorbyt, bs10900R, 1:5000), and voltage-dependent anion-selective channel protein 1 (VDAC, Abcam, ab154856, 1:5000). GAPDH in the supernatant was chosen as the internal control for free tubulin, and VDAC in the Triton X-100 insoluble fraction was chosen as the control for polymerized tubulin. Whole cell lysates (2 × 10^6^ cells) and spinal cord tissues (epicenter ± 5 mm) were prepared and analyzed. The cells and tissue homogenates were lysed for 1 h and centrifuged at 15,000*g* for 15 min at 4 °C. The content of protein was detected by BCA TM assay kit (Dalian Meilun Biology Technology Co., Ltd., China). Equal amounts of protein were loaded into the wells of the 10% SDS-PAGE gel. About 20 or 50 μg of total protein from cell or tissue homogenates were loaded into each well of the SDS-PAGE gel, respectively. The cell or tissue homogenates were separated and transferred to PVDF membranes (Millipore, USA). The membranes were blocked in Tris-buffered saline and Tween 20 (TBST) containing 5% non-fat skimmed milk for 2–3 h at room temperature. The following antibodies were used for immunoblotting: vascular endothelial growth factor A (VEGFA, Abcam, ab1316, 1:200), VEGF receptor 2 (VEGFR2, Abcam, ab39256, 1:1000), platelet-derived growth factor-B (PDGFB, Abcam, ab178409, 1:1000), PDGF receptor β (PDGFRβ, Abcam, ab32570, 1:1000), and angiopoietin-1 (Ang-1, Abcam, ab183701, 1:10000), and Tie-2 (Novus, NBP2-20637, 1:500). As a loading control, GAPDH was probed and normalized. The membranes were incubated with above mentioned antibodies for 12–14 h at 4 °C. The immunocomplexes were determined by incubating with appropriate horseradish peroxidase-conjugated secondary antibodies for 1 h at room temperature and visualized using enhanced chemiluminescence (ECL).

### Animals

Female Sprague–Dawley rats weighing 240–250 g (Pengyue Laboratory Animal Breeding Co. Ltd. Jinan, China) were housed under standard 12-h light/dark condition and received food ad libitum. All procedures involving experimental animals were approved by Ethics Committee for Animal Care and Use of Binzhou Medical University in China and were performed in accordance with the Chinese Association for Laboratory Animals Sciences.

Rats were randomly arranged to the following three groups: (a) Sham + vehicle (1:1 mixture of dimethyl sulfoxide (DMSO) and saline) group (sham group, *n* = 54), (b) SCI +vehicle (1:1 mixture of DMSO and saline) group (vehicle group, *n* = 54), and (c) SCI + Epo B group (*n* = 54). Rats were deeply anesthetized by an intraperitoneal injection of 10% chloral hydrate and underwent dorsal complete transection at the T9-10 vertebra. The animals in sham group were only subjected to laminectomy. The rats received an intraperitoneal injection of Epo B at 0.75 mg/kg (Dalian Meilun Biology Technology Co., Ltd., China) or vehicle at 1 h and 15-day post injury (Ruschel et al. 2015). The spinal cord tissues were collected at 1-day, 2-day, 7-day, and 21-day post injury. The rats were perfused transcardially with saline and rinsed thoroughly until no more blood flew out of the right atrium, followed by the fixative 4% paraformaldehyde. Spinal cords were dissected, snap frozen, and transversely sectioned at 20 μm on a cryostat. The spinal cord tissues spanning 1 mm (injury center) and spanning 4–5 mm (distal injury) rostrocaudal from the transection sites were observed, respectively.

### Evaluation functional vessels in SCI

Lycopersicon esculentum binds to and labels endothelial cells and therefore identifies perfused microvessels with an endothelial lining (Benton et al. 2009; Han et al. 2010; Muradov et al. 2013). Dylight 488-Lycopersicon Esculentum (lectin, Vector, Cat#DL-1174; Vector Laboratories, Burlingame, CA; 400 μg) was delivered systematically by retrobulbar injection 25 min prior to transcardial perfusion with saline for sacrificing the rats. The vessel function was evaluated by examining the lectin, which was circulated and bound to the vessel basal membrane and co-localized with the endothelial cell marker, rat endothelial cell antigen-1 (RECA-1), in spinal cord sections.

### Immunofluorescence staining

The section slides were warmed at room temperature for 30 min and washed in PBS for 30 min. The sections were permeabilized in 1% or 0.7% Triton X-100/PBS for 15 min and blocked in 10% normal goat serum/PBS for 4–5 h at room temperature. Antibodies against RECA-1 (Abcam, ab9774, 1:200), PDGFRβ (Abcam, ab32570, 1:100), and PODXL (podocalyxin like) Biorbyt, bs1345R, 1:50) were used as primary antibodies. The cells cultured on coverslips were fixed in 4% paraformaldehyde for 30 min, permeabilized with 0.1% Triton X-100 in PBS for 25 min, and blocked in 10% fetal bovine serum for 1.5 h. The primary antibody used was anti-α-tubulin (Abcam, ab7291, 1:100). The sections and cells were incubated in secondary antibodies for 1 h at 37 °C and imaged using laser scanning confocal microscopy.

### Cell viability

Cell Counting Kit-8 assay (CCK-8, Dalian Meilun Biology Technology Co., Ltd., China) was performed to calculate cell viability. Endothelial cells and pericytes were seeded into 96-well (1 × 10^4^cells/well) flat bottom plates with medium without Epo B (control) or medium containing different concentrations of Epo B (10 ρM, 100 ρM) for 24 h. The cells were washed for three times in PBS and incubated under the OGD condition for 60 min (OGD 60 min). CCK-8 reagents (10 μL) were added to each well, and the cells continued to culture for another 1.5 h in incubator. The optical density (OD) at 450 nm was detected by a microplate reader.

### Flow cytometry analysis of cell cycle and apoptosis

The endothelial cells and the pericytes were incubated with Epo B (10 ρM, 100 ρM) for 24 h. The cells were washed three times in PBS and incubated under the OGD condition for 60 min. The endothelial cells and pericytes (5 × 10^6^ cells) were washed in PBS, harvested (1000 g, 5 min), and fixed with ice-cold ethanol overnight at 4 °C. The cells were then washed in PBS, centrifuged at 2000*g* for 10 min, and stained with the propidium iodide (PI) and RNase A (500 μL, PI∶RNase A = 1∶9) for 30 min in the dark. The cell cycle was determined by flow cytometer (BD Biosciences). Apoptosis induction in endothelial cells and pericytes was analyzed by Annexin V-FITC apoptosis kit I (KeyGEN Biotech, KGA105). The cells (5 × 10^6^ cells) were harvested (1000*g*, 5 min) and washed in PBS followed by treatment with 500 μL binding buffer. Annexin V-FITC solution (5 μL) and PI (5 μL) were added and incubated for 15 min at room temperature. Flow cytometry was used to analyze the cells.

### Cell proliferation assay

The endothelial cells and the pericytes (2 × 10^7^ cells) were incubated with Epo B (10 ρM, 100 ρM) for 24 h. 5-Ethynyl-2′-deoxyuridine (EDU; Click-iT^TM^ EDU Flow Cytometry Assay Kit, Invitrogen^TM^, OR USA) was added to endothelial cells and pericytes at a final concentration of 50 μM and 40 μM, and the cells were incubated for further 2 h and 6 h, respectively. The cells were washed three times in PBS and incubated under the OGD condition for 60 min. Then cells (2 × 10^7^) were harvested (1000*g*, 5 min), washed (1% BSA in PBS, 3 ml), fixed (Click-iT^TM^ fixative, 100 μL), permeabilized (10% Click-iT^TM^ saponin-based permeabilization and wash reagent in PBS), and incubated with Click-iT^TM^ Plus reaction cocktail (10 μL copper protectant, 2.5 μL fluorescence dye picolylazide, 50 μL reaction buffer additive, and 435 μL PBS) according to the manufacturer’s instructions for the Click-iT^TM^ EDU Flow Cytometry Assay Kit. Flow cytometry was performed to analyze cell proliferation.

### Transwell assay

The microtubule stabilization effect on migration of endothelial cells and pericytes was evaluated using transwell chambers with an 8-μm pore-sized polycarbonate filter membrane. In the Epo B group, the cells were incubated with Epo B (10 ρM, 100 ρM) for 24 h. Then cells were trypsinized and suspended in serum-free medium. The cells (1 × 10^5^) were seeded on the upper side of chamber with the serum-free medium containing Epo B (10 ρM, 100 ρM). The lower well was filled with normal medium containing Epo B (10 ρM, 100 ρM). After being incubated for 24 h, the cells on the upper side of the membrane were gently removed. Then the cells that had migrated through the 8 μm pores were fixed with 4% paraformaldehyde and stained with crystal violet (Dalian Meilun Biology Technology Co., Ltd., China). The migrated cells were counted in five different fields under microscope and quantified by image J software (NIH, Bethesda, MD, USA).

### Angiogenesis in vitro

The in vitro angiogenesis in Matrigel assay was used to assess the spontaneous formation of capillary O-like structures by endothelial cells under normal and Epo B (10 ρM) containing conditions. The endothelial cells were treated with Epo B or medium alone for 24 h in 6-well plates until they reached more than 90% confluency. Then cells were trypsinized and suspended in serum-free medium. Before endothelial cells (2 × 10^4^ cells/100 μL) were seeded in 96-well plates containing serum-free media, the plates were coated with growth factor-reduced Matrigel matrix (40 μL, BD, Bioscience, San Jose, CA, USA) and incubated for 30 min at 37 °Cin 5% CO_2_ atmosphere. Once endothelial cells were added to the 96-well plates, they were incubated for 4 h, and tube formations were determined in four random fields from each well. The branch points and tube length were analyzed by Image J software (NIH, Bethesda, MD, USA).

### Statistical analysis

Quantitative assessments of fluorescence intensity, the number of cells migrated in transwell assay, and the band intensity value in western blotting was obtained using Image J. Immunofluorescence images were taken at the dorsal horn or central canal of the spinal cord. Data were shown as mean ± SD. The data were analyzed by SPSS version 19.0 statistic software package. One-way analysis of variance (ANOVA) analysis was used to compare three or four groups, while Student *t* tests were used for two groups. *p* < 0.05 was considered significance.

## Results

### Epo B stabilizes microtubules of OGD conditioned endothelial cells and pericytes

**Fig. 1:**
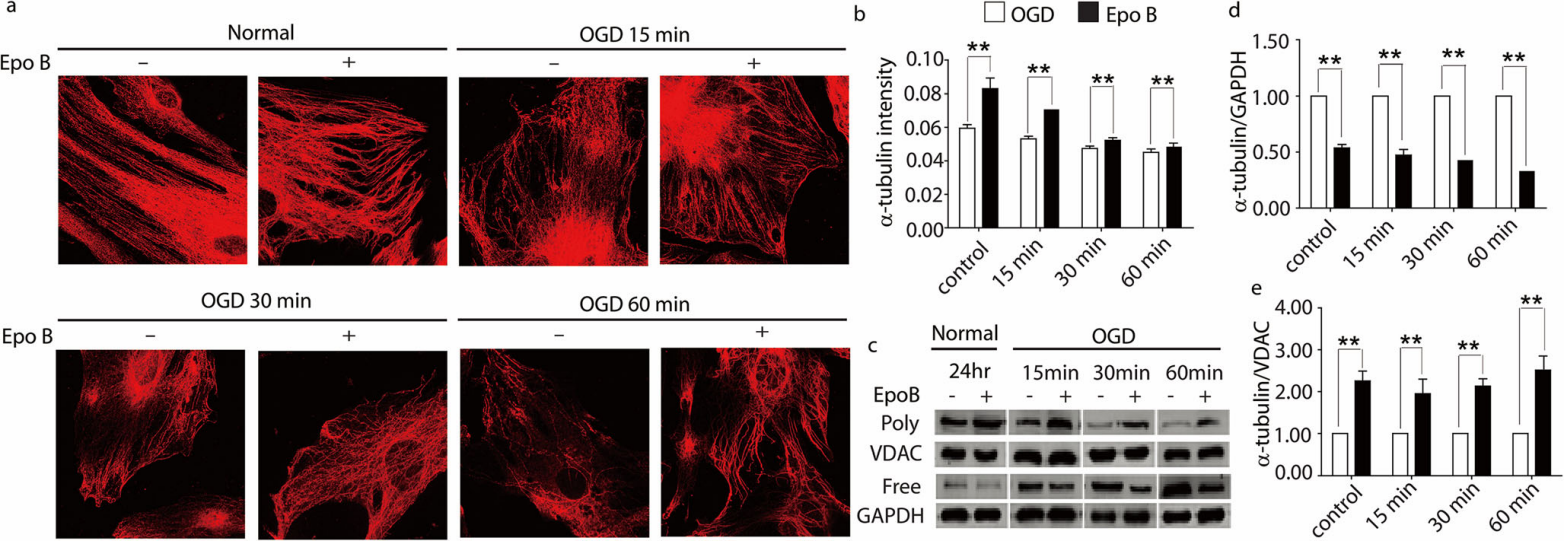
Epo B stabilizes microtubule of OGD conditioned endothelial cells. **a** Immunofluorescence staining of α-tubulin in endothelial cells. Scale bar 6 μm. **b** Statistical analysis of the fluorescence intensity of α-tubulin in endothelial cells. **c** Immunoblotting analysis of expression of free and polymerized tubulin in endothelial cells. **d–e** Statistical analysis of the relative intensity of free and polymerized tubulin in endothelial cells. The data represents the mean ± SD (*n* = 6). ** *p* < 0.01. Significance was determined by Student *t* tests.

To determine the effects of Epo B on the microtubules of OGD conditioned endothelial cells and pericytes, α-tubulin was examined with immunological methods. In quiescent endothelial cells and pericytes, the microtubules organized into a clear, uniformly, and relatively regular distributed lattice network, and Epo B-treated quiescent cells displayed more orderly organized microtubules (Figs. 1a and S1a). After these cells were subjected to OGD, the organization of the tubulins was disrupted and more disarranged. Moreover, the disruption of microtubules was time-dependent (Figs. 1a and S1a). After being treated with Epo B, the microtubule structure of both the endothelial cells and pericytes was more intact, with increased regularity and upregulated immunostaining intensity, and the microtubule organization recovered to the quiescent condition (*p* < 0.01, *n* = 6; Figs. 1b and S1b). We also detected the expression of free tubulins and polymerized tubulins in the OGD conditioned endothelial cells and pericytes by western blotting. The level of polymerized tubulin was strongly increased at all time points in both the OGD conditioned endothelial cells and pericytes treated with Epo B (*p* < 0.01, *n* = 6; Figs. 1c, e and S1c, e). In addition, the increased free tubulin caused by OGD was reversed by Epo B treatment (*p* < 0.01, *n* = 6; Figs. 1c, d and S1c, d). These results suggest that Epo B can stabilize the microtubules of endothelial cells and pericytes.

### Microtubule stabilization promotes vasculature regeneration after SCI

**Fig. 2:**
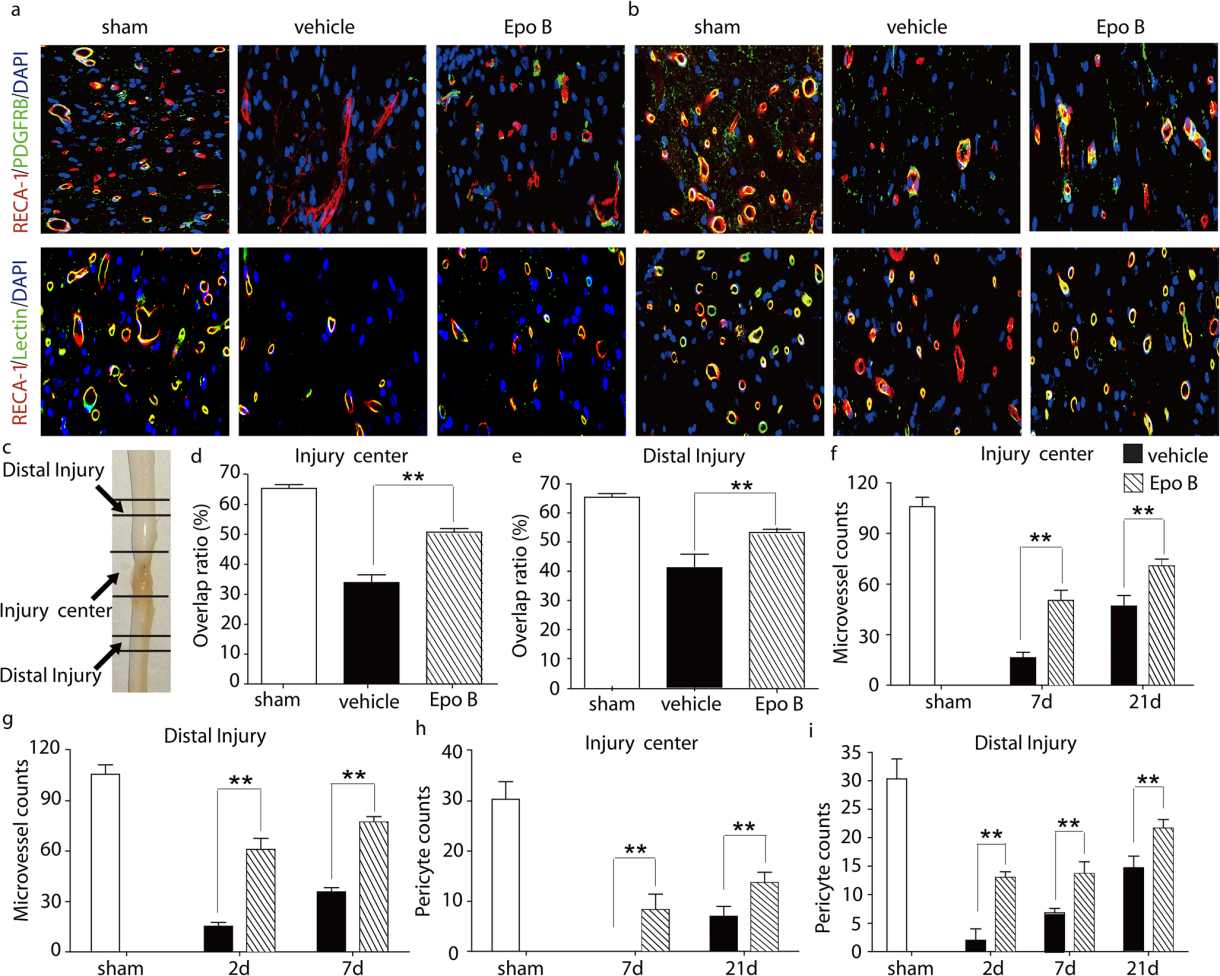
Microtubule stabilization promotes the microcirculation reconstruction after SCI. **a** Immunofluorescence staining of RECA-1, PDGFRβ, and lectin in injury center of SCI rats. Scale bar: 25 μm. **b** Immunofluorescence staining of RECA-1, PDGFRβ, and lectin in distal injury of SCI rats. Scale bar: 25 μm**. c** The definition of injury center and distal injury sites**. d–e** Statistical analysis of the relative intensity of double staining of RECA-1 and lectin in SCI rats after 21-day post injury. **f–g** Statistical analysis of the number of microvessels positive for RECA-1 in center and distal injury in SCI rats. **h–i** Statistical analysis of numbers of positive PDGFRβ expressing cells in center and distal injury sites in SCI rats. Data experiments are expressed as mean ± SD (*n* = 6). ** *p* < 0.01. Significance is determined by one-way ANOVA followed by LSD comparison tests.

To uncover the effect of microtubule stabilization on functional microvasculature after SCI, the dual staining for the injected Dylight 488-lectin and RECA-1 was performed at 21-day post injury. Our results showed that the double labeling of RECA-1 and lectin in the Epo B group was significantly increased compared with that in the vehicle group (*p* < 0.01, *n* = 6; Fig. 2a–e). There was a 1.50-fold increase of overlap ratio of RECA-1 and lectin in the injury center in the Epo B group compared with that in the vehicle group (*p* < 0.01, *n* = 6; Fig. 2a, c, d). In the distal injury site, the overlap ratio in the Epo B group was increased by 1.33-fold compared with that in the vehicle group (*p* < 0.01, *n* = 6, Fig. 2b, c, e). Our results indicate that microtubule stabilization promotes the recovery of the functional blood vessels.

To explore the mechanisms underlying the improvement in the microvessel circulation, the role of endothelial cells and pericytes in microvessel formation under the microtubule stabilization condition after SCI were examined separately. Firstly, to examine the response of the endothelial cells to microtubule stabilization, the vascular endothelial marker, RECA-1, and the lumenized vessels marker, PODXL (Li et al. 2017a), were examined and quantified with immunofluorescent staining in the central and distal sites of the injury. One-way ANOVA analysis showed that the number of microvessels positive for RECA-1 in the injury center in the Epo B group was significantly higher than that in the vehicle group, with a 3.08-fold increase at 7-day post injury and 1.50-fold increase at 21-day post injury, respectively (*p* < 0.01, *n* = 6; Fig. 2a, c, f; Table. 1). The number of microvessels positive for RECA-1 in the distal injury site in the Epo B group increased by 3.98-fold at 2-day post injury and 2.17-fold at 7-day post injury compared with that in the vehicle group (*p* < 0.01, *n* = 6; Fig. 2b, c, g; Table. 1). To reveal the lumenogenesis after SCI, double immunofluorescence staining for PODXL and RECA-1 was used to detect the lumenized vessels. In the distal injury site at 1-day, 2-day, 7-day, and 21-day post injury, the overlap ratio of RECA-1 and PODXL was 18.01 ± 2.34%, 0.30 ± 0.14%, 16.84 ± 6.33%, and 20.75 ± 4.83%, respectively, in the vehicle group, and 22.84 ± 3.35%, 0.47 ± 0.35%, 18.05 ± 3.53%, and 21.38 ± 3.80%, respectively, in the Epo B group (data not shown). The overlap ratio in the Epo B group was higher than that in the vehicle group, but there was no significant difference (*p* > 0.05, *n* = 6). Secondly, to examine response of the pericytes to microtubule stabilization, the pericyte marker, PDGFRβ along with the endothelial cell marker, RECA-1, was investigated. In the injury center region, the PDGFRβ immunopositive cells in the Epo B group were 8.33 ± 3.05 cells/mm^2^ at 7-day post injury and 13.67 ± 1.52 cells/mm^2^ at 21-day post injury, which were significantly higher than those in the vehicle group (*p* < 0.01, *n* = 6; Fig. 2a, c, h; Table. 1). In the distal injury region, the PDGFRβ immunopositive cells in the Epo B group were increased by 6.40-fold at 2 day, 2.00-fold at 7 day, and 1.48-fold at 21-day post injury compared with those in the vehicle group. The PDGFRβ labeled cells were significantly increased in the Epo B group (*p* < 0.01, *n* = 6; Fig. 2b, c, i; Table. 1). In summary, the immunoreactivities of both the endothelial marker, RECA-1, and pericyte marker, PDGFRβ were upregulated in the SCI model with microtubule stabilization treatment, thereby illustrating that stabilized microtubule treatment may promote the restoration of microvessels in SCI rats.

**Table 1:** Microtubule stabilization promotes the reconstruction of vasculature after SCI

Time post injury (days)	RECA-1 (cells/mm^2^)		PDGFRβ (cells/mm2)	
	Vehicle group	Epo B group	vehicle group	Epo B group
	Count^*^	Count	Count^**^	Count
	Injury center			
7	16.33 ± 3.21	50.33 ± 6.08	0.00 ± 0.00	8.33 ± 3.05
21	47.00±6.25	70.67 ± 4.04	7.00±2.00	13.67 ± 2.08
	Distal injury			
2	15.33 ± 2.31	61.00 ± 6.55	2.00 ± 2.00	13.00±1.00
7	35.67 ± 2.52	77.33 ± 3.21	6.83 ± 0.77	13.67±2.08
21	94.67±6.02	97.00 ± 5.29	14.67 ± 2.18	21.67 ± 1.52

*RECA-1 antibody immunohistochemistry labeled vessel numbers in sham experiment were 100.17 ± 2.47cells/mm^2^

**PDGFRβ immunohistochemistry labeled cell numbers in sham experiment were 30.33 ± 3.51cells/mm^2^

### Microtubule stabilization promotes cell proliferation and inhibits apoptosis but not lumenogenesis

**Fig. 3:**
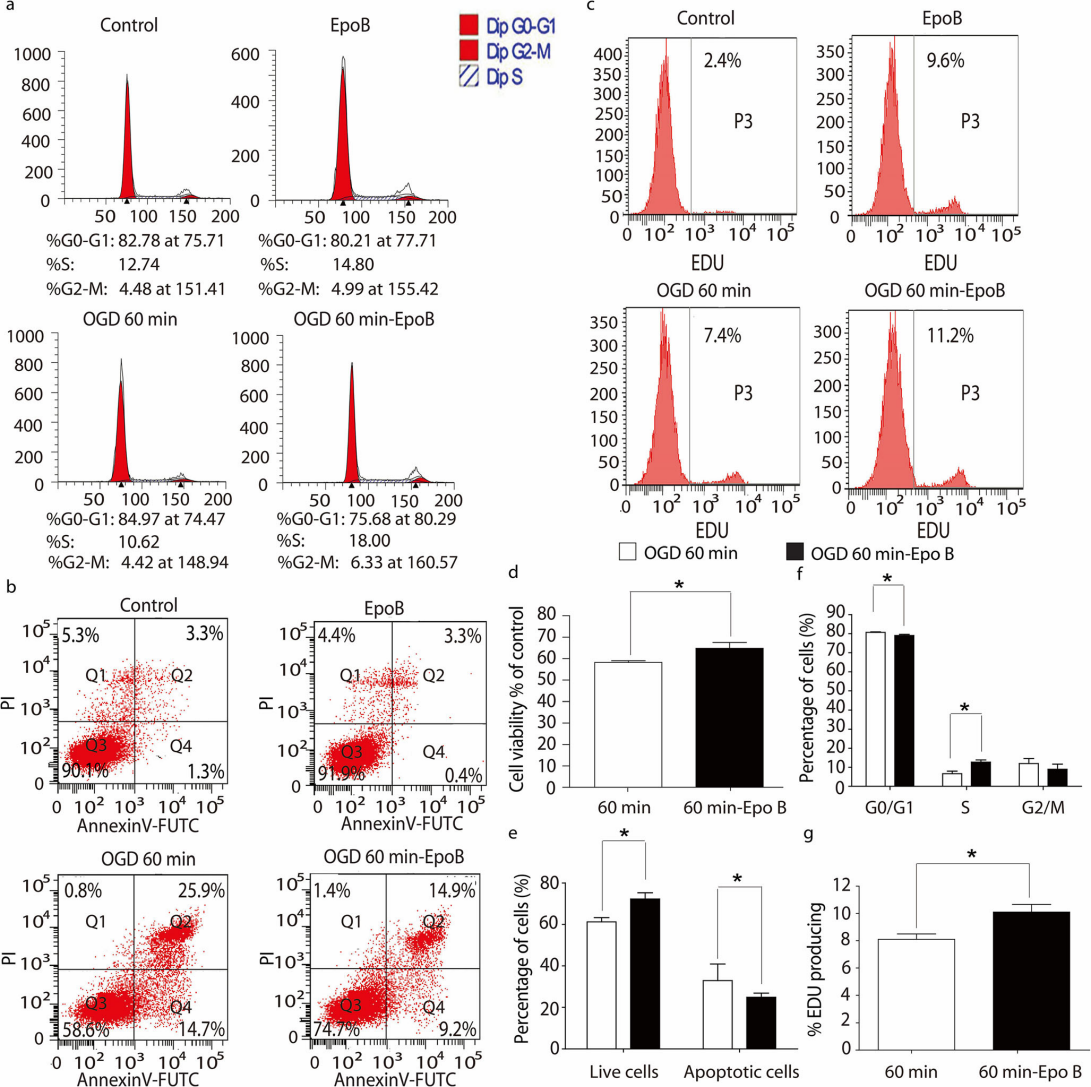
Microtubule stabilization promotes cell proliferation and inhibits apoptosis in endothelial cells. **a** The cell cycle of endothelial cells in each group was determined by flow cytometry. **b** The endothelial cells apoptosis in each group was tested by flow cytometry. **c** The fluorescence intensity of EDU expressing endothelial cells in each group was measured by flow cytometry. **d** The viabilities of endothelial cells were analyzed by CCK-8 assay. **e** Statistical analysis of the number of apoptosis of endothelial cells. **f** Statistical analysis of the number of endothelial cells in each phase in each group. **g** Statistical analysis of the fluorescence intensity of EDU expressing endothelial cells in each group. Results are expressed as mean ± SD (*n* = 6). * *p* < 0.05. Significance is determined by student t-tests.

To explore the mechanisms underlying the promotion of microcirculation reconstruction after SCI by microtubule stabilization, first, the proliferation of endothelial cells and pericytes was examined by CCK-8 and flow cytometry. According to the data above presented, the OGD 60 min was chosen for following experiments. The CCK-8 assay indicated that the viability of the endothelial cells and pericytes in the OGD 60 min conditioned endothelial cells and pericytes treated with Epo B (OGD 60-min Epo B group) was significantly higher than that in the OGD 60 min group (*p* < 0.05, *n* = 6; Figs. 3d and S3d). The percentage of endothelial cells in the resting G0/G1 phase was 80.51 ± 1.51% in the OGD 60-min Epo B group, which was significantly lower than that in the OGD 60 min group (84.67 ± 0.37%, *p* < 0.05, *n* = 6; Fig. 3a, f). The percentage of endothelial cells in the proliferative S phase was 13.53±1.11% in the OGD 60-min Epo B group, which was significantly higher than that in the OGD 60-min group (10.66 ± 0.56%, *p* < 0.05, *n* = 6; Fig. 3a, f) The percentage of pericytes in the proliferative S phase in the OGD 60-min Epo B group was significantly higher than that in the OGD 60-min group (*p* < 0.05, *n* = 6; Fig. S3a, f). In the OGD 60-min Epo B group, the percentage of EDU labeled proliferative endothelial cells and pericytes were 10.10±0.96% and 27.27 ± 5.31%, respectively (Figs. 3c, g and S3c, S3g); whereas in the OGD 60-min group, they were 8.10 ± 0.69% and 17.96 ± 2.25%, respectively (Figs. 3c, g and S3c, g). The percentage of EDU labeled proliferative cells in the OGD 60-min Epo B group was significantly higher than that in the OGD 60 min group (*p* < 0.05, *n* = 6; Figs. 3c, g and S3c, g). These results indicate that the microtubule stabilization treatment promotes both the endothelial cells and pericytes from the G0/G1 phase to S phase.

Second, the apoptosis of endothelial cells and pericytes was also examined. In comparison to the OGD 60-min group, the number of live cells in the OGD 60-min Epo B group was significantly increased (Figs. 3b and S3b). In the OGD 60-min Epo B group, the apoptotic cells were significantly decreased compared with those in the OGD 60-min group (*p* < 0.05, *n* = 6; Figs. 3b, e and S3b, e).

Third, we also detected the effect of Epo B on lumenogenesis. The endothelial lumen formation is the first step in microcirculation regeneration. The endothelial tube formation in vitro was also quantified by counting the branch points and tube length. The branch points were 402.21 ± 15.93 branches/mm^2^ in the Epo B group and 388.50 ± 24.68 branches/mm^2^ in the control group. The number of branch points in the Epo B group was higher than that in the control group (increased by 1.03-fold). The tube length in the Epo B group was increased by 1.08-fold compared with that in the control group. However, there was no significant difference between these two group (*p>*0.05, *n* = 6; Fig. S4), which was consistent with the results in vivo. These results suggest that Epo B could not promote lumenogenesis. Together these results suggest that microtubule stabilization promotes cell proliferation and inhibits apoptosis but does not promote lumenogenesis.

### Microtubule stabilization inhibits the migration of endothelial cells and pericytes

**Fig. 4:**
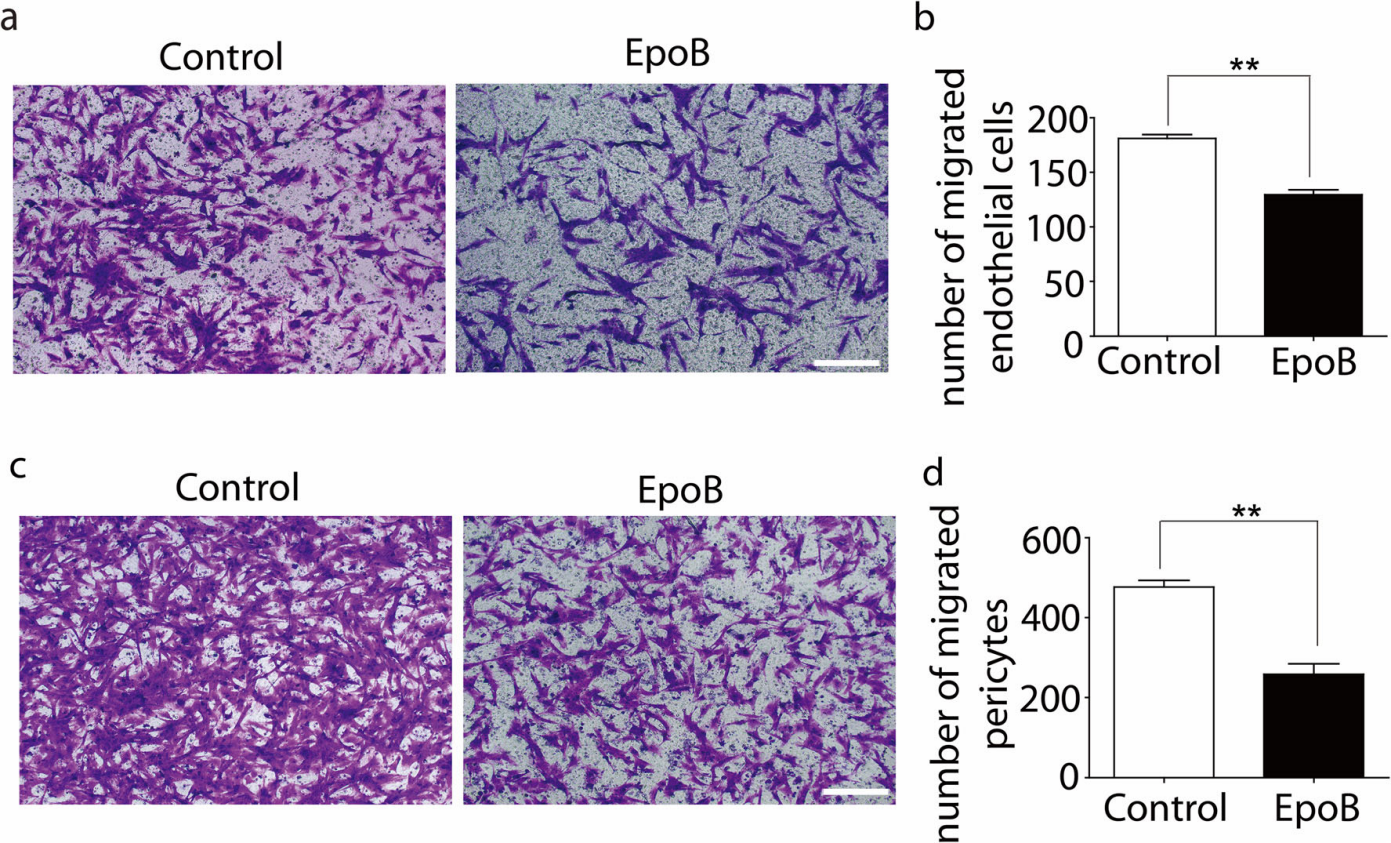
Microtubule stabilization inhibits the migration of endothelial cells and pericytes. **a** Crystal violet staining of migrated endothelial cells. Scale bar: 25μm. **b** Statistical analysis of migrated numbers of endothelial cells. **c** Crystal violet staining of migrated pericytes. Scale bar: 25 μm. **d** Statistical analysis migrated numbers of pericytes. Results were expressed as mean ± SD (*n* = 6). ** *p* < 0.01. Significance was determined by student t-tests.

To examine the migration response of endothelial cells and pericytes to microtubule stabilization, the transwell experiment was performed. The results showed that the migration of both endothelial cells and pericytes was significantly inhibited by Epo B. The number of migrated endothelial cells was 181.10 ± 6.06 cells/mm^2^ in the control group and 129.50±7.89 cells/mm^2^ in the Epo B group (*p* < 0.01, *n* = 6; Fig. 4a–b). The migrated pericytes were 476.60±28.65 cells/mm^2^ in the control group and 257.90 ± 45.70 cells/mm^2^ in the Epo B group (reduction by 45.89%, *p* < 0.01, *n* = 6; Fig. 4c–d). The results revealed that the migration of endothelial cells and pericytes was suppressed by microtubule stabilization.

### Microtubule stabilization upregulates expression of the angiogenesis related proteins

**Fig. 5:**
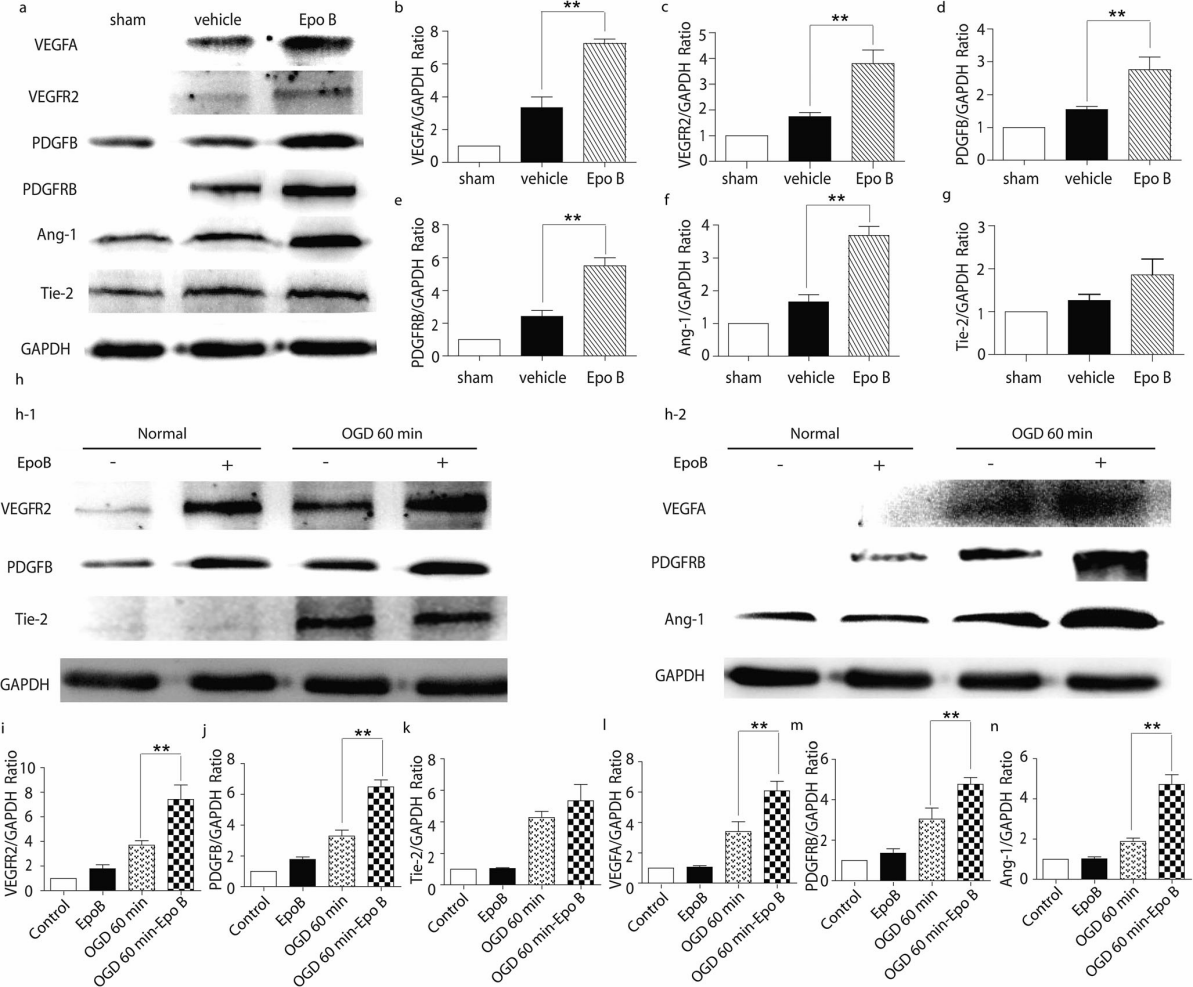
Microtubule stabilization upregulates the expression of angiogenesis related proteins. **a** Immunoblotting analysis of the expressions of VEGFA, VEGFR2, PDGFB, PDGFRβ, Ang-1 and Tie-2 in three groups of SCI rats. **b–g** Statistical analysis of the relative intensity of VEGFA, VEGFR2, PDGFB, PDGFRβ, Ang-1, and Tie-2 in SCI rats. **h–1** Immunoblotting analysis of the expressions of VEGFR2, PDGFB, and Tie-2 in endothelial cells in four groups. **h–2** Immunoblotting analysis of the expressions of VEGFA, PDGFRβ, and Ang-1 in pericytes in four groups. **i–k** Statistical analysis of the relative intensity of VEGFR2, PDGFB, and Tie-2 in endothelial cells in four groups. **l-n** Statistical analysis of the relative intensity of VEGFA, PDGFRβ, and Ang-1 in pericytes in four groups. Results are expressed as mean ± SD (*n* = 6). ** *p* < 0.01. Significance is determined by one-way ANOVA followed by LSD comparison tests.

VEGFA, VEGFR2, PDGFB, PDGFRβ, Ang-1, and Tie-2 are essential in the initiation, formation, and functioning of blood vessels. To examine the effect of microtubule stabilization treatment on the expression of angiogenesis related molecules in vivo, the tissue obtained from SCI rats at 21-day post injury was collected and examined with western blotting. The results demonstrated that the expressions of VEGFA, VEGFR2, PDGFB, PDGFRβ, and Ang-1 in the Epo B group were significantly increased by 2.16-, 2.00-, 1.79-, 2.28, and 2.23-fold compared with those in the vehicle group (*p* < 0.01, *n* = 6; Fig. 5a–f). The expression of Tie-2 in the Epo B group was increased by 1.42-fold but was not significantly different compared with that in the vehicle group (*p>*0.05, *n* = 6; Fig. 5g).

To examine the effects of microtubule stabilization treatment on the expression of angiogenesis related molecules in vitro, the expressions of VEGFA, VEGFR2, PDGFB, PDGFRβ, Ang-1, and Tie-2 in endothelial cells and pericytes under the OGD condition treated with or without Epo B were examined. Even that OGD alone increase angiogenesis related proteins (Freitas-Andrade et al. 2008; Gao et al. 2020; Ulleras et al. 2001), the expressions of all these six proteins were more upregulated in both endothelial cells and pericytes treated with Epo B. Similar to the results from the SCI rats, the expressions of VEGFA, VEGFR2, PDGFB, PDGFRβ, and Ang-1 were significantly upregulated in the OGD 60-min Epo B group compared with those in the OGD 60 min group (*p* < 0.01, *n* = 6; Fig. 5h–j and l–n). Consistent with the results of the in vivo experiments, the expression of Tie-2 showed no significant difference between the OGD 60-min and the OGD 60-min Epo B group (*p>*0.05, *n* = 6; Fig. 5k). These data reveal that VEGFA, VEGFR2, PDGFB, PDGFRβ, and Ang-1 may be involved in the process of restoration of the microvessels induced by microtubule stabilization treatment.

## Discussion

Our study identifies a novel mechanism by which microtubule stabilization exerts protective effects against microvasculature dysfunction in SCI. Our findings suggest that microtubule stabilization protects microcirculation by inhibiting the apoptosis and migration of endothelial cells and pericytes and promoting proliferation of endothelial cells and pericytes, which may be related to the upregulation of VEGFA, VEGFR2, PDGFRB, PDGFRβ, and Ang-1.

The destruction of microcirculation after SCI leads to ischemia and hypoxia in the local injury site, which contributes to neuron apoptosis, astrocyte necrosis, and scar formation. These serious pathological and physiological changes create a microenvironment which is unfavorable for nerve regeneration. Microcirculation reconstruction is one of the key factors of nerve regeneration. The major components of microvessels are endothelial cells and pericytes (Armulik et al. 2005). Various physiological activities of cells, such as cell division, proliferation, apoptosis, necrosis, and migration are affected by altering the microtubule structure and expression. Epo B is a microtubule stabilizing anti-cancer drug, which promotes the polymerization of tubulin heterodimer into microtubule polymers (Altmann 2003). It is demonstrated that the microtubules of scar-forming fibroblasts and neurons were stabilized by Epo B. However, the effect of Epo B on endothelial cells and pericytes still remains unclear. Our study showed OGD induced breaking and fragmentation of microtubules of endothelial cells and pericytes in a time-dependent manner, but after treatment with Epo B, the endothelial cells and pericytes were more stabilized, organized, and exhibited less disruption of the microtubules. Therefore, in this study, we focused on the effects of microtubules stabilization induced by Epo B on the microcirculation reconstruction after SCI.

Microtubules are one of the components of the cytoskeleton. Microtubules are involved in cell migration, organelles transportation, and chromosome segregation during mitosis and cell fate determination (Goodson and Jonasson 2018). Microtubule disruption can lead to a series of adverse consequences, such as insufficient transportation of intracellular material, disturbance in the formation of intercellular gap junction and the dysfunction of mitochondria, which result in irreversible damage of the cells (Devillard et al. 2006). Stabilization of the microtubules with taxol has been suggested to protect against hypoxia/re-oxygenation injury, while the microtubule depolymerizer, colchicine, has been reported to abolish the protective effect of ischemic pre-conditioning (Hu et al. 2010). The above-mentioned physical activities orchestrated by microtubule are essential in initiation, formation, and maturation in angiogenesis. Endothelial cells are the main component of the microvasculature and are responsible for establishing direction and developing tubular structures. Pericytes then invade the newly vascularized tissues and localize at the growing front of the endothelial sprouts. Pericytes ensure that the newly formed vessel is mature, functional and stabilized by regulating the vesicle transport and bulk flow transcytosis, upregulating the endothelial tight junction protein, and moderating the tight junctional alignment (Jo et al. 2013; Nehls et al. 1992). In this study, the pericytes and microvessels, especially functionally perfused microvessels, were increased by microtubule stabilization treatment. Furthermore, the motor function of hind limbs of SCI rats was significantly recovered after microtubule stabilization treatment (Fig. S2). Microtubule stabilization is required for formation and maintenance of angiogenic structures (Bayless and Johnson 2011). A stable microtubule network in endothelial cells secures vesicle trafficking to apical membranes to provide nutrition and drive vessel formation (de Forges et al. 2012; Norden et al. 2016). The inflammation-induced endothelial barrier dysfunction was rescued by microtubule stabilization after acute lung injury (Li et al. 2015). In addition, the stabilized microtubule in pericytes maintained microvasculature function (Li et al. 2017b). Some studies have shown that the loss of pericytes leads to endothelial hyperplasia and dysfunction and the coverage of pericytes in microvessels contributes to the vasculature regeneration (Hellstrom et al. 2001; Lu et al. 2019). Therefore, once the vasculature establishes, the microtubule network must remain intact and stabilized to maintain a regular angiogenic network (de Forges et al. 2012). Taken together, microtubule stabilization treatment could promote microcirculation reconstruction, which is favorable for axon regeneration to improve functional recovery after SCI.

Lumenogenesis is an initial and fundamental process in microcirculation regeneration. It has been reported that the stable microtubules of endothelial cells promote lumenogenesis and sufficient tissue reperfusion after ischemic injury by upregulating expression of acetylated and detyrosinated tubulins (Kim et al. 2013; Li et al. 2017a). However, lumenogenesis was not affected by microtubule stabilization in this study as our results revealed that the acetylated tubulin in endothelial cells was not upregulated by microtubule stabilization treatment (Data not shown). This result could be explained by the use of Epo B, which only modifies the structure of microtubule, but does not alter the acetylation of microtubules. Furthermore, during lumen formation, endothelial cells are separated into tip cells and stalk cells. The former is responsible for sprouting and establishing direction, while the latter proliferate, migrate, follow the tip cells, and differentiate to form the lumen (Duran et al. 2017). Our results indicated that the migration of endothelial cells was inhibited by microtubule stabilization treatment. The physical cell migration is induced by a very precise regulation of microtubule dynamic at the leading edge, and the microtubule catastrophe is also essential at the cells‘ leading edges (Drabek et al. 2006; Niethammer et al. 2004). Both are required to maintain a rapid turnover of focal adhesions, which allows for rapid and efficient migration (de Forges et al. 2012). However, in this study, the microtubule was stabilized by Epo B, which inhibited the microtubule catastrophe at the cells’ leading edges, and consequently inhibited the cell migration. Collectively, our results suggested that the microtubule stabilization treatment may promote microvascular regeneration via the proliferation of stalk cells.

VEGFA, VEGFR2, PDGFB, PDGFRβ, Ang-1, and Tie-2 play an essential role in microcirculation reconstruction. VEGFA is expressed in pericytes and its receptor, VEGFR2, is expressed in endothelial cells (Duran et al. 2017). Their interaction promotes cell survival and proliferation via increasing the expression of anti-apoptotic Bcl-2 and survivin (Sweeney et al. 2016). Consistent with this, our research showed that VEGFA and VEGFR2 were up-regulated in the Epo B-treated group in vivo and in vitro and the apoptosis of endothelial cells was inhibited by microtubule stabilization treatment. However, the survival and proliferation of endothelial cells were increased by microtubule stabilization treatment. In addition, combined VEGF and PDGFB treatment reduces secondary degeneration by promoting angiogenesis after SCI (Lutton et al. 2012). VEGFA binding to VEGFR2 upregulates PDGFB expression in pericytes, thereby promotes binding to its receptor, PDGFRβ. The binding of these molecules facilitates proliferation and inhibits apoptosis. Consistent with those studies, in this study, PDGFB and PDGFRβ were upregulated in the Epo B-treated group in vivo and in vitro, the apoptosis of pericytes was decreased, and their survival and proliferation were promoted by microtubule stabilization. Activation of the PI3K pathway by PDGFB-PDGFRβ promotes actin reorganization, stimulates cell growth, and reduces cell apoptosis (Hu et al. 1995). PDGFB- or PDGFRβ-null mice die at mid-gestation from cardiovascular defects. These embryos show abnormal angiogenesis and their blood vessels have poorly organized basement membranes and decreased coverage of pericytes (Armulik et al. 2005). In vivo, our results showed that the loss of pericytes coverage was decreased by microtubule stabilization at 2 day post injury. In vitro, we demonstrated that the migration of pericytes was inhibited by microtubule stabilization. Moreover, the interaction of the above molecules upregulated Ang-1 in pericytes through a cascade of reactions with its receptor Tie-2, which is expressed in endothelial cells (Jain 2003). Their interaction acts on plus end proteins to facilitate microtubule polymerization, which is indispensable for vessel stabilization and maturation (Armulik et al. 2005; Sweeney et al. 2016). This study demonstrated that Ang-1 was upregulated in the Epo B-treated group in vivo and in vitro, but Tie-2 was not upregulated. Recently, Savant S et al. have indicated that Tie-1 counter-regulated Tie-2 cell surface presentation and sustained Tie-2 signaling and cell survival in remolding of endothelial cells (Savant et al. 2015). The expressions of orphan receptor Tie-1 are increased in tip cells, which results in the elevated Tie-2 being neutralized. This explains why Tie-2 was not enhanced by microtubule stabilization treatment. The specific mechanisms need to be investigated further. Importantly, the results from this study demonstrated that VEGFA, VEGFR2, PDGFB, PDGFRβ, and Ang-1 are involved in the effect of microtubule stabilization on microvessel regeneration after SCI.

In this study, we demonstrated that microtubule stabilization protects microvessels and promotes the restoration of microcirculation. This study provides evidence of the potential use of microtubule stabilization as a therapeutic target to reduce microcirculation dysfunction after SCI in the clinic. In addition, it indicates a novel theoretical basis for the tissue engineering, such as adding to microtubule stabilizers or removing of microtubule destabilizers.

## Electronic supplementary material

ESM 1 (DOCX 7031 kb)
