# Supplementary material for: Microtubule Stabilization Promotes Microcirculation Reconstruction After Spinal Cord Injury
Source: J Mol Neurosci. 2020 Sep 8;71(3):583–95. doi: 10.1007/s12031-020-01679-5 (PMC7851021; doi:10.1007/s12031-020-01679-5)
Supplement: Supplementary file 1 — (DOCX 7031 kb) [file 12031_2020_1679_MOESM1_ESM.docx]

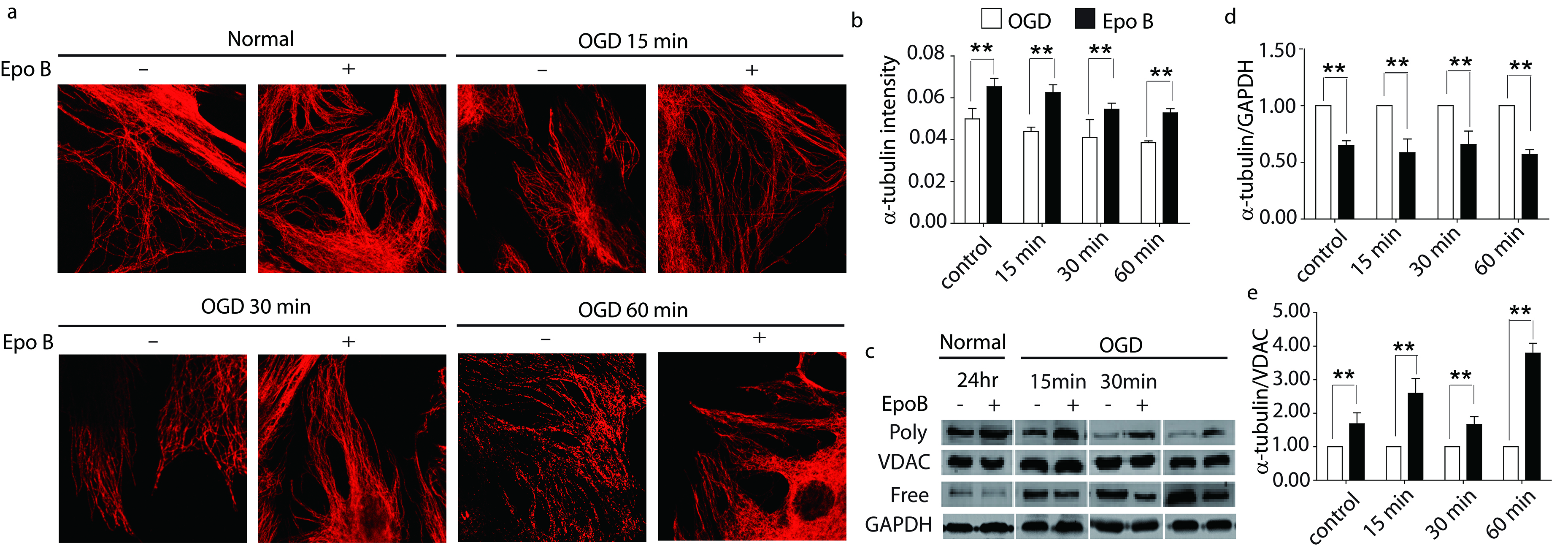


**Fig.S1** Epo B stabilizes microtubule of OGD conditioned pericytes. **a** Immunofluorescence staining of α-tubulin in pericyte. Scale bar 6 μm. **b** Statistical analysis of the fluorescence intensity of α-tubulin in pericytes. **c** Immunoblotting analysis of expression of free and polymerized tubulin in pericytes. **d-e** Statistical analysis of the relative intensity of free and polymerized tubulin in pericytes. The data represents the mean $\pm$± SD (n=6). **p*<0.05, ***p*<0.01. Significance was determined by student t-tests.


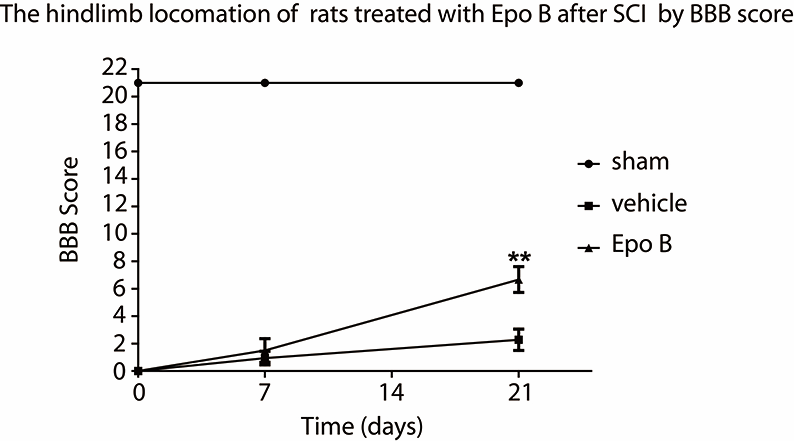


**Fig.S2** Microtubule stabilization promotes the recovery of the motor function of hind limbs after SCI. Statistical analysis of Basso-Beaie-Bresnehan (BBB) score. The data represents the mean $\pm$± SD (n=9). ***p*<0.01. Significance was determined by student t-tests.


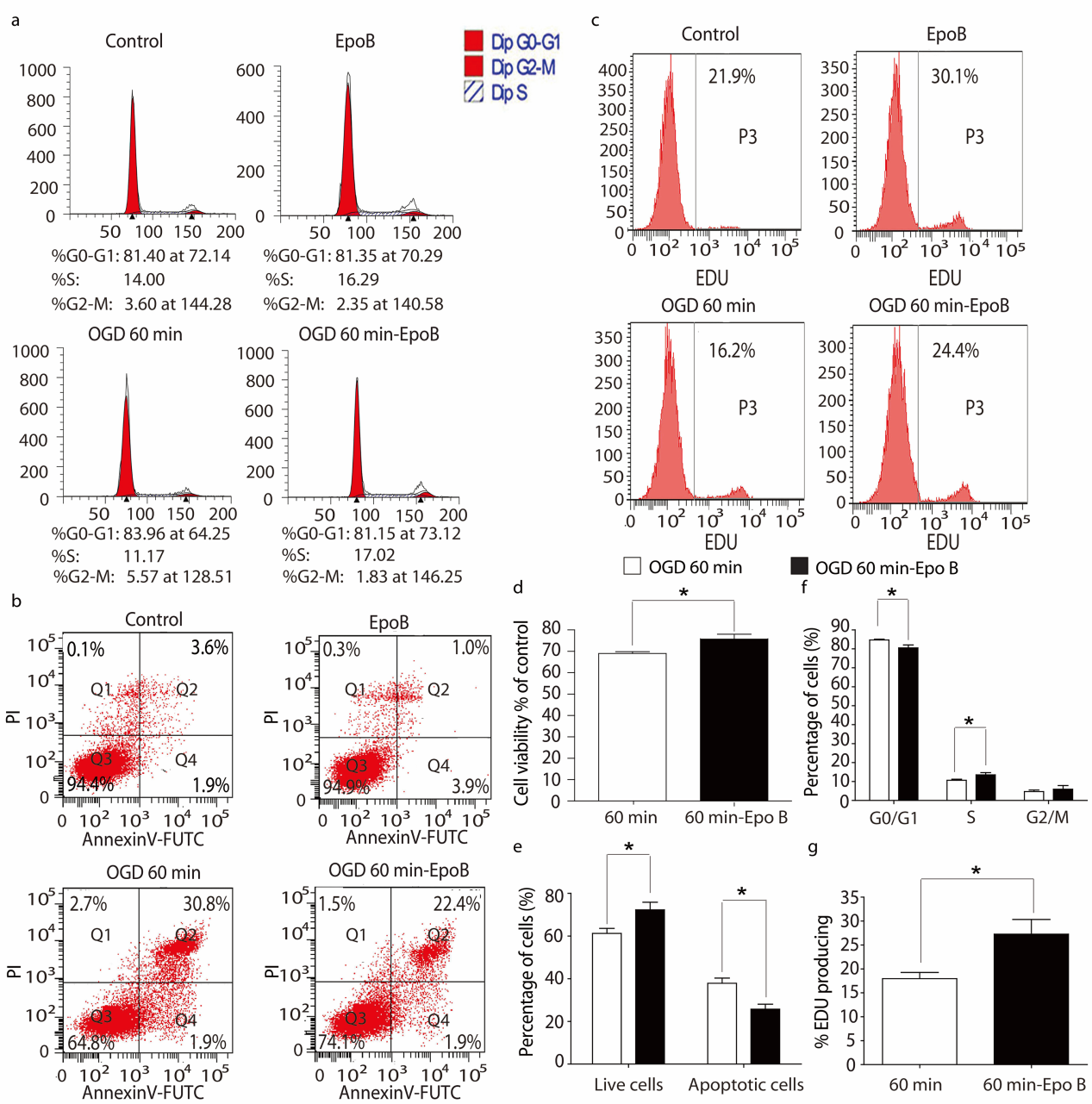


**Fig.S3** Microtubule stabilization promotes cell proliferation and inhibits apoptosis in pericytes. **a** The cell cycle of pericytes in each group was determined by flow cytometry. **b** The pericytes apoptosis in each group was tested by flow cytometry. **c** The fluorescence intensity of EDU expressing pericytes in each group was measured by flow cytometry. **d** The viabilities of pericytes were analyzed by CCK-8 assay. **e** Statistical analysis of the number of apoptosis of pericytes. **f** Statistical analysis of the number of pericytes in each phase in each group. **g** Statistical analysis of the fluorescence intensity of EDU expressing pericytes in each group. Results are expressed as mean $\pm$ $\pm$± SD (n=6). **p*<0.05. Significance is determined by student t-tests.


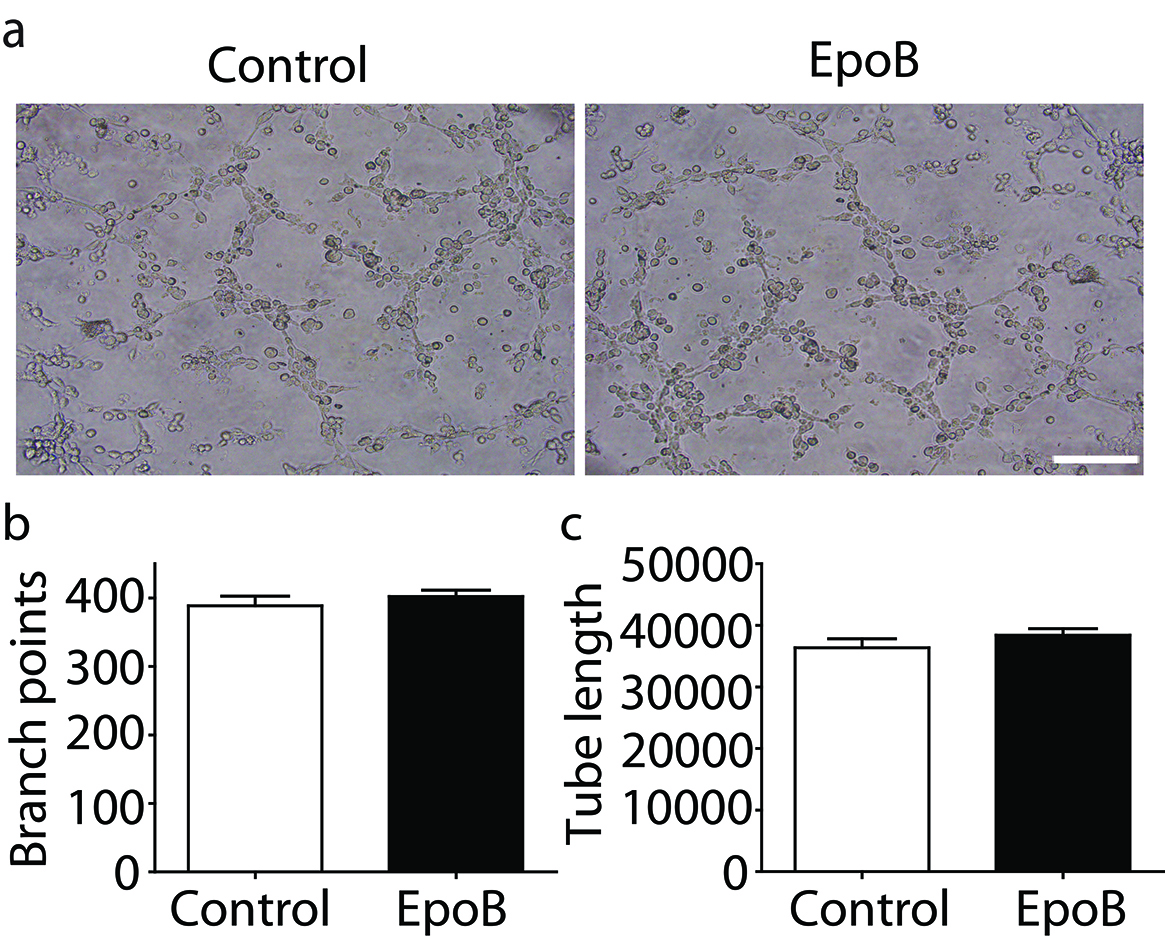


**Fig.S4** Microtubule stabilization does not promote lumenogenesis of endothelial cells. **a** The lumenogenesis was determined by cell tube formation assay. **b** Statistical analysis of the branch points. **c** Statistical analysis of the tube length. Results are expressed as mean $\pm$± SD (n=6). *p*>0.05. Significance is determined by student t-tests.
